# Quality of Life and Societal Costs Related to Celiac Disease Before and After Diagnosis

**DOI:** 10.14309/ctg.0000000000000965

**Published:** 2025-12-19

**Authors:** Anna L. Heilig, M. Elske van den Akker-van Marle, Floris van Overveld, Caroline Meijer-Boekel, M. Luisa Mearin, Jan M. Heijdra Suasnabar

**Affiliations:** 1 Department of Biomedical Data Science, Leiden University Medical Center, Leiden, the Netherlands;; 2 Dutch Celiac Society, Naarden, the Netherlands;; 3 Department of Pediatric Gastroenterology, Leiden University Medical Center, Willem Alexander Children's Hospital, Leiden, the Netherlands.

**Keywords:** celiac disease, quality of life, diagnostic process, health economics

## Abstract

**INTRODUCTION:**

Celiac disease (CD) may affect quality of life (QoL), health care use, and societal costs in various ways both before and after diagnosis. However, detailed evidence remains limited about how costs and QoL change after diagnosis and which patient characteristics (e.g., symptom duration) influence those changes. The aim of this study was to evaluate CD patients' QoL and societal costs before and after diagnosis.

**METHODS:**

In this cross-sectional study, 2,691 patient-members of the Dutch Celiac Society completed a questionnaire about their life before and after diagnosis. Data collection included sociodemographic and clinical characteristics, health care use, non–health care costs, and QoL (measured using the EuroQol 5-Dimension 5-Level). Mean QoL and annual health care and societal costs were compared prediagnosis and postdiagnosis. Multivariate regression models were used to identify factors associated with QoL and costs in both periods (and difference between periods).

**RESULTS:**

On average, respondents recalled experiencing 4–5 symptoms before diagnosis. QoL improved significantly postdiagnosis, with greater improvements among childhood diagnoses. Annual health care and societal costs decreased by 23% and 36%, respectively, postdiagnosis, except for societal costs in those diagnosed during childhood. Age at diagnosis and the number of prediagnosis symptoms were associated with lower and higher recalled prediagnosis QoL, respectively. Number of symptoms, comorbidities, and nonadherence to a gluten-free diet were associated with lower postdiagnosis QoL.

**DISCUSSION:**

After CD diagnosis, QoL significantly improved and health care and societal costs decreased, except for societal costs among childhood diagnoses. These findings enhance the understanding of CD's burden and economic impact, supporting health care and policy efforts for timely CD identification.

## Graphical abstract

**Figure:**
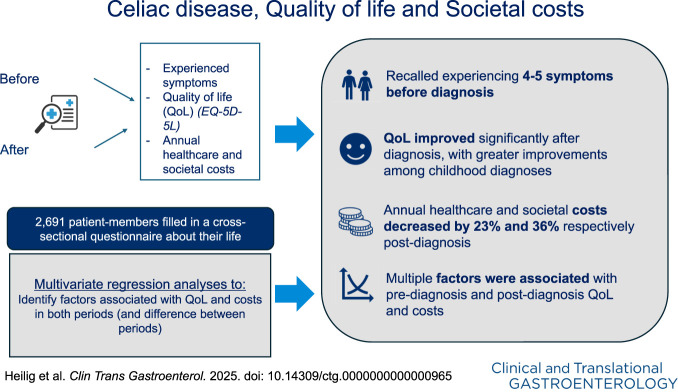

## INTRODUCTION

Celiac disease (CD) is a genetic autoimmune disorder that is characterized by intestinal inflammation and damage resulting from an inappropriate immune response to gluten ingestion (1). The prevalence of CD varies and is generally estimated to be around 1% worldwide (2). Over the past 2 decades, detection and diagnosis rates of CD have increased (1). However, despite this improved detection and the development of new diagnostic tools, millions of people still have undiagnosed and consequently untreated CD (3). Of the diagnoses that do occur, many occur years after the onset of symptoms, primarily because of the fact that CD often presents with a broad range of nonspecific intestinal and extraintestinal symptoms (4). Owing to the high variability of symptoms associated with CD, the European Society for Paediatric Gastroenterology, Hepatology, and Nutrition classification was developed, which categorizes CD into symptomatic, asymptomatic, and potential CD (5).

The quality of life (QoL) of people with CD is negatively affected by these intestinal and extraintestinal symptoms (6,7). Even after diagnosis and adherence to a gluten-free diet (GFD), which is currently the only effective treatment for CD, the impact on patients' QoL is complex. Some studies suggest that adherence to a GFD normalizes QoL, while others suggest that it changes QoL but does not normalize it (8–10). A persistent impact of CD on QoL after diagnosis may be related to the dietary and related social restrictions imposed by a GFD (11–13). Moreover, the persistence of symptoms despite strict adherence to a GFD may also negatively affect patients' QoL (6).

Besides the restrictive nature of a GFD, the high cost of gluten-free products has also been reported as a barrier to GFD compliance (14). When examining the costs associated with a disease, the societal perspective has been emphasized, which is important in the context of CD (15). This societal perspective considers all societal actors and related costs, regardless of who bears them, including health care costs as well as those borne by patients, families, and other sectors (16). From this perspective, the economic burden of CD also includes productivity losses (including absenteeism or presenteeism) and costs related to GFD adherence (15). A 2022 study showed that people with CD experience greater productivity losses before and after diagnosis than the general population (17). From a health care perspective, studies have shown that the healthcare utilization and costs of CD diagnosis increase in the year before diagnosis (18–20). The impact of CD diagnosis on these costs after diagnosis has been inconsistent. A study in the United States found that health care utilization and costs directly decreased in the year after CD diagnosis (19). By contrast, in the United Kingdom and Sweden, health care utilization and costs initially increased after CD diagnosis, but decreased in the long term without fully normalizing (18,20). However, information on the impact of CD diagnosis on costs from a societal perspective is scarce because studies adopting this perspective are limited and difficult to extrapolate to different settings (15). In addition, only a few studies have compared the QoL before and after diagnosis, and to our knowledge, none have investigated this in the Dutch setting.

This study aims to assess the QoL and societal costs of patients before and after being diagnosed with CD in the Netherlands. By evaluating these factors, this study aims to provide insight into the impact of CD on patients and Dutch society.

## METHODS

### Study design and population

This study used a cross-sectional survey design. Participants were invited to complete an online questionnaire through an advertisement on the website of the Dutch Celiac Society (in Dutch: Nederlandse Coeliakie Vereniging). Data collection took place between October and December of 2022. Individuals were eligible for this study if they had a self-reported medically confirmed diagnosis of CD and were fluent in Dutch. Respondents who completed the entire questionnaire were included in the analyses. For participants younger than 18 years, the questionnaire could be completed by themselves or by a proxy (parent or guardian). All included participants provided informed consent before survey completion, and data collection was approved by the Medical Ethics Committee of Leiden-Den Haag-Delft (P17.240/NL63291.058.17) as part of a larger registered project (Landelijk Trial Register, number NL7089).

### Survey

The online questionnaire consisted of 33 questions collecting information on sociodemographic (e.g., sex, age), clinical characteristics of the participants (e.g., duration of symptoms, year of diagnosis), as well as costs and QoL before and after diagnosis. For prediagnosis questions, participants were asked to recall the period before their diagnosis, and for postdiagnosis questions, they were asked to consider their current situation or the past year. Supplementary Questionnaire 1 (Supplementary Digital Content 1, http://links.lww.com/CTG/B443) contains the full questionnaire completed by the respondents.

The Dutch version of the EuroQol 5-Dimension 5-Level (EQ-5D-5L) was used to measure QoL prediagnosis and postdiagnosis (21,22). This instrument measures health-related QoL across 5 dimensions (i.e., mobility, self-care, usual activities, pain/discomfort, and anxiety/depression) on a 5-point scale, ranging from “no problems” (1) to “extreme problems/unable to” (5) (23,24). Corresponding utility scores (i.e., where 1 = perfect health and 0 = as bad as death) were also calculated using the tariffs of the general Dutch population (25). In addition, the EQ-Visual Analog Scale (VAS) was also included, which ranges from 0 (“Worst imaginable health state”) to 100 (“Best imaginable health state”) (26).

The societal perspective is adopted for costs, which encompasses both the health care–related and non–health care-related costs (27). The cost-related questions collected information on health care utilization (e.g., general practitioner [GP], dietitian consultations) and productivity losses (e.g., absenteeism from work) prediagnosis and postdiagnosis, as well as costs of a GFD postdiagnosis. These societal costs were calculated by summing these costs. For the postdiagnosis period, patients reported their annual health care utilization patterns, while for their prediagnosis period, they reported their total health care utilization (i.e., over their undiagnosed period). The monetary costs of health care utilization and productivity losses were calculated using published Dutch reference prices (16). The reference price for outpatient visits (€120.00) was used for all health care visits, except for dietician visits (€43.31) (16). Productivity losses were calculated using a cost per hour of €39.88, assuming a maximum work loss of 115 days, which corresponds to the established friction period in the Netherlands (16). All costs are expressed in 2023 Euros, adjusted from 2022 values using the consumer price index (28). Costs for serological tests (tissue Transglutaminase Immunoglobulin A and Endomysial Immunoglobulin A) were based on Procedures File (in Dutch: Verrichtingenbestand) (29).

### Statistical analysis

Descriptive statistics were used to summarize patient demographic, clinical, and cost data. The dependent sample *t* test was used to compare QoL, annual health care utilization and costs, and annual societal costs prediagnosis and postdiagnosis. The independent sample *t* test was used to compare differences in symptoms experienced by age group. As CD characteristics (e.g., symptoms and resource use) are known to differ between children and adults, descriptive analyses and statistical comparisons were stratified by age at diagnosis (diagnosed as children, i.e., younger than 18 years and those diagnosed as adults, i.e., 18 years or older) (30).

To compare *annual* health care utilization prediagnosis and postdiagnosis, patients' total prediagnosis health care utilization was annualized by dividing over their reported duration of symptoms (in years) until diagnosis. Moreover, a diagnostic test and diagnostic visit (GP and gastroenterologist) was assumed for respondents (N = 313) who did not report either one because this is the minimum required diagnostic process described in Dutch CD guidelines (31).

Multivariate linear regression was used to assess the contribution of various factors to QoL, annual health care costs, and annual societal costs before, after, and the difference between before and after a CD diagnosis. Prediagnosis analyses included as predictors: sex, age at diagnosis, number and duration of prediagnosis symptoms, and comorbidities. In addition, GFD adherence was included in the postdiagnosis analyses and difference analyses. Assumptions of linearity and collinearity were tested (the latter using Pearson correlations with a threshold of > 0.60, reported in Supplementary Table 1, Supplementary Digital Content 1, http://links.lww.com/CTG/B443). Multivariate analyses were restricted to respondents who reported QoL or cost-related data for both the prediagnosis and postdiagnosis periods. All statistical analyses were performed with SPSS version 29.

## RESULTS

### Characteristics of study population

Out of 3,241 respondents to the survey, a total of 2,691 fully completed the questionnaire and were included in this study. Approximately one-fifth of the respondents were diagnosed with CD during childhood (before age 18). On average, those diagnosed in childhood (61%) were more likely than those diagnosed in adulthood (52%) to have no family history of CD. Nearly all respondents self-reported adherence to a GFD postdiagnosis (99%) (Table 1).

**Table 1.** Descriptive statistics of the respondents

Variables	Overall	Childhood diagnoses (<18 yr)	Adult diagnoses (≥18 yr)
N (%)	2,691 (100)	596 (22)	2,095 (78)
Age at survey (yr), mean (SD)	44.7 (21.2)	17.3 (11.2)	52.6 (16.3)
Age at diagnosis (yr), mean (SD)	34.4 (19.5)	6.7 (5.1)	42.2 (14.2)
Time between diagnosis and survey completion (yr), mean (SD)	10.3 (9.9)	10.6 (11.3)	10.4 (9.5)
Sex, n (%)			
Male	634 (24)	159 (27)	475 (23)
Female	2,050 (76)	435 (73)	1,615 (77)
Other	7 (0.3)	2 (0.3)	5 (0.2)
Family diagnosed with CD, n (%)			
No CD within family	1,457 (54)	361 (61)	1,096 (52)
≥1 family with CD	1,050 (39)	210 (35)	840 (40)
I do not know	184 (7)	25 (4)	159 (8)
Self-reported GFD adherence, n (%)			
Always	2,668 (99)	592 (99)	2,076 (99)
Sometimes	9 (0.3)	2 (0.3)	7 (0.3)
Rarely/never	14 (0.5)	2 (0.4)	12 (0.6)
Endoscopy and biopsies at diagnosis, n (%)	2,103 (78)	282 (47)	1,821 (87)
Comorbidity, n (%)^a^	1,405 (52)	195 (33)	1,210 (58)
No. of symptoms/signs, median (IQR)	4.7 (2.4)	4.0 (2.0)	4.9 (2.5)
Duration of symptoms/signs (yr), mean (SD)	9.8 (3.2)	2.7 (3.2)	11.9 (14.3)

CD, celiac disease; GFD, gluten-free-diet; IQR, interquartile range.

a Comorbidity = co-occurrence of multiple disease in 1 respondent.

On average, respondents reported experiencing 4–5 symptoms before their diagnosis, with the mean duration of these symptoms being more than 4 times longer in those diagnosed during adulthood (11.9 years) compared with those diagnosed during childhood (2.7 years).

### Prevalence of symptoms

**Figure 1.**
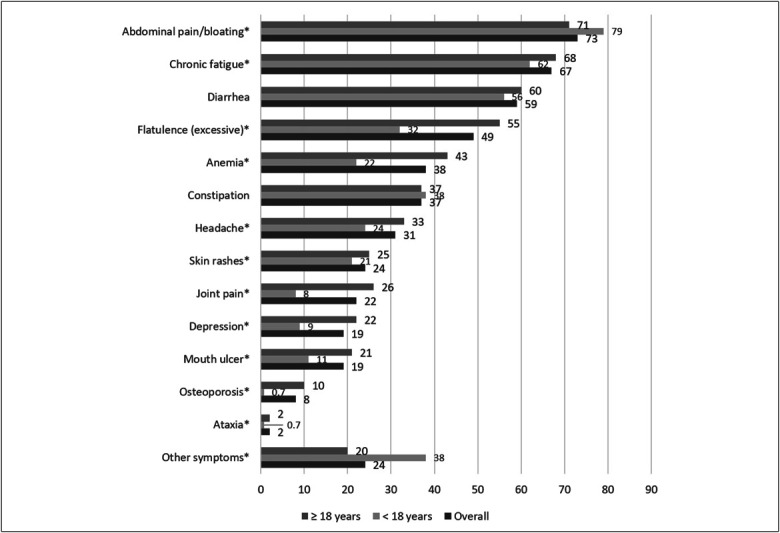
Prevalence of recalled symptoms/signs before celiac disease diagnosis^1^. ^1^Sample size: 2,691; *Significant difference between 18-year or older group and younger than 18-year group (*t* test *P* value ≤ 0.05).

Nearly all (98%) of respondents recalled experiencing at least 1 symptom/sign prediagnosis of CD (Supplementary Table 2, Supplementary Digital Content 1, http://links.lww.com/CTG/B443). The distribution of reported prediagnosis symptoms by age at diagnosis is shown in Figure 1. The most common symptoms reported were abdominal pain/bloating (73%), chronic fatigue (67%), and diarrhea (59%). Patients diagnosed as adults were on average more likely to report excessive flatulence, anemia, mouth ulcers, joint pain, and osteoporosis compared with those diagnosed during childhood, who were more likely to report abdominal pain/bloating and other symptoms.

### QoL before and after CD diagnosis

Table 2 shows the respondents' QoL data (i.e., EQ-5D-5L, EQ-utility and EQ-VAS) of the recalled prediagnosis and the reported postdiagnosis, stratified by age at diagnosis. The distributions of responses for each EQ-5D-5L domain are shown in Supplementary Table 3 (Supplementary Digital Content 1, http://links.lww.com/CTG/B443).

**Table 2.** Mean score per QoL domain, EQ-utility, and EQ-VAS before^a^ and after CD diagnosis (n = 2,685)

QoL domain	Before CD diagnosis, mean (SD)	After CD diagnosis, mean (SD)	Difference: after − before, mean (95% CI)
Mobility^b^			
Overall	1.35 (0.83)	1.24 (0.62)	−0.11 (−0.14 to −0.08)*
Childhood diagnoses	1.53 (1.12)	1.06 (0.32)	−0.47 (−0.55 to −0.38)*
Adult diagnoses	1.30 (0.71)	1.29 (0.68)	−0.01 (−0.04 to 0.02)
Self-care^b^			
Overall	1.16 (0.65)	1.06 (0.32)	−0.11 (−0.13 to −0.08)*
Childhood diagnoses	1.45 (1.13)	1.05 (0.37)	−0.41 (−0.49 to −0.32)*
Adult diagnoses	1.08 (0.38)	1.06 (0.30)	−0.02 (−0.04 to −0.01)**
Usual activities^b^			
Overall	2.16 (1.19)	1.49 (0.81)	−0.68 (−0.72 to −0.63)*
Childhood diagnoses	2.41 (1.30)	1.29 (0.65)	−1.12 (−1.22 to −1.01)*
Adult diagnoses	2.09 (1.15)	1.54 (0.84)	−0.55 (−0.60 to −0.50)*
Pain/discomfort^b^			
Overall	2.72 (1.21)	1.69 (0.83)	−1.03 (−1.08 to −0.98)*
Childhood diagnoses	3.01 (1.26)	1.49 (0.69)	−1.52 (−1.62 to −1.41)*
Adult diagnoses	2.64 (1.18)	1.75 (0.85)	−0.89 (−0.94 to −0.84)*
Anxiety/depression^b^			
Overall	2.00 (1.15)	1.52 (0.79)	−0.49 (−0.53 to −0.45)*
Childhood diagnoses	1.99 (1.18)	1.50 (0.83)	−0.49 (−0.58 to −0.40)*
Adult diagnoses	2.01 (1.15)	1.52 (0.78)	−0.49 (−0.53 to −0.44)*
EQ-5D-5L utility^c^			
Overall	0.64 (0.30)	0.83 (0.16)	0.20 (0.19 to 0.21)*
Childhood diagnoses	0.56 (0.35)	0.86 (0.14)	0.30 (0.28 to 0.33)*
Adult diagnoses	0.66 (0.28)	0.83 (0.17)	0.17 (0.16 to 0.18)*
EQ-VAS^d^			
Overall	54.30 (22.37)	78.76 (15.89)	24.47 (23.50 to 25.43)*
Childhood diagnoses	47.18 (24.22)	84.13 (14.02)	36.95 (34.82 to 39.07)*
Adult diagnoses	56.33 (21.39)	77.24 (16.05)	20.91 (19.88 to 21.94)*

CD, celiac disease; CI, confidence interval; EQ-5D-5L, EuroQol 5-Dimension 5-Level; EQ-VAS, EQ Visual Analogue Scale; QoL, quality of life.

a Participants reported recalled quality of life.

b 1 = no problems to 5 = extreme problems/unable to.

c 1 = perfect health.

d 100 = perfect health.

*Significant (*P* < 0.001); **significant (*P* < 0.01) difference between before and after CD diagnosis.

Respondents diagnosed in childhood reported more problems across most EQ-5D dimensions compared with those diagnosed in adulthood, except for the anxiety/depression dimension. Respondents reported significantly improved QoL postdiagnosis in all dimensions except mobility for those diagnosed in adulthood compared to their recalled prediagnosis QoL. Respondents diagnosed as children showed greater mean improvement in mobility, self-care, usual activities, and pain/discomfort compared with the improvements among those diagnosed during adulthood. Those diagnosed during childhood had a higher mean reported improvement for the EQ-utility and EQ-VAS of 0.13 and 16.04, respectively, compared with those diagnosed during adulthood.

### Health care and societal costs

Health care utilization and costs decreased significantly postdiagnosis compared with recalled prediagnosis utilization and costs, except for dietitian consultations in those diagnosed during childhood (Table 3). Overall, annual health care costs decreased by 23% postdiagnosis, with a higher mean decrease for those diagnosed in adulthood (44%) vs in childhood (14%). The largest contributor to reduced health care utilization and costs was GP visits, which accounted for 65% of the reduction in frequency and 55% of the reduction in cost compared with recalled prediagnosis.

**Table 3.** Annual health care costs and societal costs: health care resource use and costs per patient before^a^ and after CD diagnosis^b^

	Before diagnosis (B), mean (SD)			After diagnosis (A), mean (SD)			Difference: after–before diagnosis, mean (95% CI)		
	B; overall	B; <18	B; ≥18	A; overall	A; <18	A; ≥18	Difference; overall	Difference; <18	Difference; ≥18
Healthcare use^c^									
Total healthcare use	10.4 (9.1)	9.0 (8.8)	10.7 (9.2)	2.5 (3.8)	2.2 (3.5)	2.5 (3.8)	−7.9 (−7.6 to −8.3)*	−6.8 (−6.0 to −7.5)*	−8.2 (−7.8 to −8.7)*
General practitioner	5.6 (5.2)	4.5 (4.7)	5.9 (5.3)	0.4 (1.3)	0.3 (1.2)	0.5 (1.3)	−5.1 (−4.9 to −5.3)*	−4.1 (−3.8 to −4.5)*	−5.4 (−5.2 to −5.6)*
Internist	1.2 (0.8)	0.5 (1.9)	1.4 (2.9)	0.2 (0.7)	0.1 (0.5)	0.2 (0.7)	−1.0 (−0.9 to −1.1)*	−0.4 (−0.2 to −0.6)*	−1.2 (−1.1 to −1.3)*
Gastroenterologist	1.7 (2.0)	1.5 (2.1)	1.7 (2.0)	0.9 (1.7)	0.5 (1.3)	1.0 (1.8)	−0.8 (−0.7 to −0.9)*	−1.0 (−0.8 to −1.2)*	−0.7 (−0.6 to −0.8)*
Dietician	0.7 (2.1)	0.4 (1.7)	0.7 (2.2)	0.5 (1.3)	0.5 (1.2)	0.6 (1.3)	−0.1 (−0.0 to −0.2)**	0.1 (0.3 to −0.0)	−0.2 (−0.1 to −0.3)*
Other	1.2 (3.3)	2.1 (3.8)	1.0 (3.0)	0.4 (1.5)	0.8 (2.0)	0.3 (1.3)	−0.9 (−0.7 to −1.0)*	−1.4 (−1.0 to −1.7)*	−0.7 (−0.6 to −0.8)*
Costs^c^									
General practitioner costs	€229 (287)	€349 (376)	€195 (246)	€72 (216)	€54 (193)	€78 (222)	€−156 (−143 to −169)*	€−295 (−262 to −329)*	€−117 (−104 to −131)*
Internist costs	€153 (474)	€111 (508)	€164 (463)	€90 (€312)	€36 (215)	€105 (333)	€−63 (−43 to −83)*	€−75 (−32 to −118)*	€−60 (−37 to −82)*
Gastroenterologist costs	€297 (440)	€396 (601)	€269 (378)	€408 (759)	€247 (571)	€454 (798)	€111 (144 to 78)*	€−149 (−87 to −218)*	€185 (220 to 146)*
Dietician costs	€90 (387)	€96 (446)	€88 (369)	€248 (594)	€237 (560)	€251 (604)	€158 (183 to 132)*	€141 (197 to 86)	€162 (191 to 133)*
Other resource costs	€182 (630)	€487 (948)	€96 (471)	€166 (688)	€356 (889)	€113 (609)	€−16 (−18 to 49)	€−131 (−31 to −231)**	€17 (49 to −15)
Healthcare costs	€1,268 (1,333)	€1,646 (1,821)	€1,161 (1,137)	€984 (1,501)	€929 (1,423)	€1,000 (1,523)	€−284 (−209 to −359)*	€−717 (−1,242 to −1,789)*	€−162 (−83 to −243)*
Productivity losses^d^	€4,645 (13,544)	€1,670 (7,677)	€5,482 (14,687)	€1,270 (7,225)	€896 (4,053)	€1,375 (7,889)	€−3,375 (−2,863 to −3,886)*	€−774 (−218 to −1,330)*	€−4,107 (−3.474 to −4.741)*
GFD costs	—	—	—	€1,507 (870)	€1,592 (864)	€1,483 (871)	€1,507 (870)	€1,592 (864)	€1,483 (871)
Non–health care costs^a^	€4,645 (13,544)	€1,670 (7,677)	€5,482 (14,687)	€2,776 (7,348)	€2,488 (4,247)	€2,858 (8.007)	€−1,868 (−1,357 to −2,380)*	€818 (1,376 to 260)**	€−2,625 (−1,992 to −3,258)*
Societal costs	€5,912 (13,664)	€3,316 (7,886)	€6,644 (14,811)	€3,760 (7,768)	€3,416 (4,652)	€3,857 (8,439)	€−2,152 (−1,631 to −2,673)*	€101 (693 to −492)	€−2,787 (−2,143 to −3,430)*

CD, celiac disease; CI, confidence interval; GFD, gluten-free diet.

a Recalled health care use and costs.

b Sample size: 2,685.

c Annualized by dividing the total over the duration of symptoms until diagnosis.

d Includes 31% respondents who did not work.

*Significant (*P* < 0.001); **significant (*P* < 0.05).

Recalled prediagnosis non–health care costs were 70% higher for those diagnosed in adulthood compared with childhood, mainly because of productivity losses accrued by undiagnosed adults.

Non–health care costs increased by 50% (€818) for those diagnosed in childhood, while these mean costs decreased significantly by 50% (€−2,625) for those diagnosed in adulthood. The GFD costs (€1,483) incurred by those diagnosed in adulthood were outweighed by the savings in lost productivity (€−4,107).

Overall, the annual societal costs per respondent were significantly lower postdiagnosis (36% lower, €2,152). Significantly lower annual societal costs were also observed for those diagnosed as adults, but higher annual societal costs were observed for those diagnosed as children.

### Factors associated with QoL and CD diagnosis

Table 4 reports the results of multivariate linear regression analyses of factors associated with QoL recalled before and reported after CD diagnosis. The number of symptoms recalled experiencing by respondents prediagnosis was significantly negatively associated with QoL prediagnosis and postdiagnosis. Conversely, age at diagnosis and prediagnosis symptom duration were both significantly positively associated with recalled prediagnosis QoL, with respondents diagnosed as adults and/or respondents with a longer prediagnosis symptom duration being associated with higher/better mean QoL. Finally, comorbidities and nonadherence to a GFD were both negatively associated with postdiagnosis QoL.

**Table 4.** Factors associated with quality of life (EQ-utility) before^a^ and after CD diagnosis

Covariates^b^	Before diagnosis			After diagnosis			Difference		
	Coefficient	95% CI	*P* value	Coefficient	95% CI	*P* value	Coefficient	95% CI	*P* value
Sex (M [ref]/F)	0.000	−0.039 to 0.003	0.087	−0.000	0.000 to 0.000	0.953	0.000	0.000 to 0.001	0.163
Age diagnosis (<18 [ref]/≥18)	0.149	0.124 to 0.175	<0.001	−0.008	−0.023 to 0.007	0.310	−0.157	−0.184 to −0.130	<0.001
No. symptoms before diagnosis	−0.061	−0.065 to −0.056	<0.001	−0.015	−0.017 to −0.012	<.001	0.046	0.041 to 0.051	<0.001
Duration symptoms till diagnosis (yr)	0.001	0.000 to 0.002	0.020	0.000	−0.001 to 0.000	0.503	−0.001	−0.002 to 0.000	0.10
Comorbidities (no [ref]/yes)	−0.018	−0.039 to 0.003	0.087	−0.060	−0.073 to −0.048	<.001	−0.042	−0.064 to −0.021	<0.001
GFD adherence (always [ref]/not)	—	—	—	−0.081	−0.143 to −0.018	0.011	−0.052	−0.164 to 0.060	0.363

CD, celiac disease; CI, confidence interval; GFD, gluten-free-diet.

a Recalled quality of life.

b Sample size: 2,680.

Table 4 also presents the factors associated with a pre-post difference in QoL. Improvements in QoL were positively associated with the recalled prediagnosis number of symptoms and significantly negatively associated with age at diagnosis and comorbidities. In other words, respondents diagnosed in adulthood or with comorbidities experienced less improvement in QoL after becoming diagnosed.

### Factors associated with health care and societal costs before and after CD diagnosis

Table 5 shows the results of multivariate linear regression analyses of variables associated with health care and societal costs before and after diagnosis. Prediagnosis health care and societal costs were significantly positively associated with the number of prediagnosis symptoms. Being diagnosed during adulthood and having a longer recalled prediagnosis symptom duration was significantly associated with lower prediagnosis annual health care costs, whereas being diagnosed as an adult was significantly associated with higher prediagnosis societal costs.

**Table 5.** Factors associated with annual healthcare and societal costs before^a^ and after CD diagnosis

Covariates^b^	Before diagnosis			After diagnosis			Difference		
	Coefficient	95% CI	*P* value	Coefficient	95% CI	*P* value	Coefficient	95% CI	*P* value
Annual health care costs									
Sex (M [ref]/F)	1	−1 to 2	0.347	0.6	−1 to 2	0.511	0	−2 to 2	0.932
Age diagnosis (<18 [ref]/≥18)	−218	−338 to −98	<0.001	−33	−180 to 114	0.656	185	−2 to 372	0.053
No. symptoms before diagnosis^a^	43	22 to 63	<0.001	61	36 to 87	<0.001	19	−14 to 51	0.260
Duration symptoms till diagnosis (yr)^a^	−37	−41 to −33	<0.001	−0.2	−5 to 5	0.947	−37	31 to 43	<0.001
Comorbidities (no [ref]/yes)	96	−1 to 192	0.053	97	−22 to 216	0.109	−1	−150 to 153	0.985
GFD adherence (always [ref]/not)	—	—	—	236	−374 to 846	0.448	−135	−643 to 912	0.734
Annual societal costs									
Sex (M [ref]/F)	4	−11 to 19	0.588	0.5	−8 to 9	0.910	4	−12 to 19	0.645
Age diagnosis (<18 [ref]/≥18)	2,216	930 to 3,501	<0.001	102	−643 to 848	0.788	−2,095	−3,412 to −777	0.002
No. symptoms before diagnosis^a^	1,258	1,034 to 1,483	<0.001	487	357 to 618	<0.001	−776	−1,006 to −546	<0.001
Duration symptoms till diagnosis (yr)^a^	−37	−79 to 5	0.084	−27	−51 to −3	0.028	9	−33 to 52	0.666
Comorbidities (no [ref]/yes)	915	−125 to 1,955	0.085	360	−243 to 964	0.241	562	−1,627 to 504	0.301
GFD adherence (always [ref]/not)	—	—	—	2,005	−1,092 to 5,101	0.204	1,827	−7,296 to 3,643	0.513

CD, celiac disease; CI, confidence interval; GFD, gluten-free diet; QoL, quality of life.

a Recalled health care use and costs and QoL.

b Sample size: 2,683; difference annual health care costs intercept: −1,010; difference annual societal costs intercept: 5,216.

For the postdiagnosis health care and societal costs, these were significantly positively associated with the recalled prediagnosis number of symptoms. Whereas, the recalled prediagnosis symptom duration was significantly negatively associated with postdiagnosis societal costs.

Table 5 also presents the factors associated with a difference in costs between patients' postdiagnosis and prediagnosis periods. The intercepts are included to facilitate the estimation of expected differences in costs for specific combinations of predictor variables. Longer recalled prediagnosis symptom duration was significantly negatively associated with the difference of healthcare costs. For the difference in annual societal costs, being diagnosed during adulthood and having more recalled pre-diagnosis symptoms were significantly associated with a decrease in the difference in societal costs.

## DISCUSSION

In this cross-sectional study, we evaluated the impact of CD on patient's QoL and societal costs comparing the retrospectively assessed before situation with their current situation. Our results show that respondents' QoL improved significantly postdiagnosis compared with recalled prediagnosis situation. Health care and societal costs decreased after CD diagnosis, except among those diagnosed during childhood.

The average EQ-5D-5L utility score postdiagnosis was 0.83, lower than the Dutch average score of 0.87 (25). This indicates that QoL does not normalize after CD diagnosis which aligns with findings from Majsiak et al, but contrasts with studies reporting QoL comparable with the general population after diagnosis (8–10,32). Our multivariate analyses showed that age at diagnosis and the recalled number of symptoms before diagnosis were, respectively, associated with higher and lower prediagnosis QoL, in line with previous findings (8). In addition, confirming previous literature, we observed a negative association between nonadherence to a GFD and QoL postdiagnosis (33,34). Nonadherence most often results in persistent symptoms and consequently reduced QoL, but other contributing factors may also play a role. For instance, food insecurity, reported by 23% of individuals with CD in the Netherlands, may contribute to both nonadherence and lower QoL because it was associated with increased barriers to GFD adherence and lower self-reported mental well-being (35). The multivariate regressions that showed significant associations representing clinically relevant differences in QoL because the observed coefficients were higher than the minimal important difference (0.037 − 0.069) of the EQ-utility (36).

Being diagnosed during adulthood and having had a longer recalled symptom duration was associated with lower prediagnosis annual health care costs, diverging from previous findings (18). A potential explanation is that older individuals, or those with a longer symptom duration, may not perceive them as significantly impairing (as longer symptom duration was associated with higher QoL), potentially delaying diagnosis and reducing healthcare use. For postdiagnosis, we observed that these annual health care costs decreased, which is consistent with the findings from the United States (19), but in contrast to findings from the United Kingdom and Sweden (18,20). These conflicting results may be due to differences in the scope of costs considered because the latter countries included reimbursement of GFD costs in the health care perspective, which was not the case in our study and the US study (18–20). Despite these differences, all studies which included GFD costs identified it as a substantial contributor to postdiagnosis costs.

Another noteworthy finding was that postdiagnosis societal costs (i.e., including GFD and productivity losses besides health care costs) decreased among adult diagnosed cases but not among childhood diagnosed cases. This discrepancy may partly reflect missing data on school absenteeism and subsequent parental work loss in childhood cases, potentially underestimating non–health care costs in this subgroup (37,38). Nonetheless, the fact that societal costs among childhood diagnoses did not significantly differ between prediagnosis and postdiagnosis does not imply that earlier CD detection is unwarranted from a cost-effectiveness perspective. A recent modeling study showed that, even with a notable increase in postdiagnosis societal costs, childhood screening and case-finding were highly cost-effective in the long-term (39).

Several limitations should be noted. First, the retrospective study design made the data susceptible to recall bias. Previous research has shown that common symptoms associated with a disease may be overestimated compared with more atypical symptoms (40).In addition, the recall period may be associated with (and therefore bias) participants' responses. We checked for this potential limitation and found only marginal associations (smaller than minimal important difference) between time because diagnosis and prediagnosis EQ-utility or prediagnosis societal costs (Supplementary File 5, Supplementary Digital Content 1, http://links.lww.com/CTG/B443). Although there was a small negative association between postdiagnosis societal costs and time since diagnosis, the overall effect of recall period on the reliability of our results appears to be limited. Second, to calculate annual health care utilization, all prediagnosis health care utilization was assumed to have occurred within the recalled symptomatic period, which is also susceptible to recall bias of respondents. Furthermore, it was assumed that each respondent underwent diagnostic test and visit. This assumption was checked by excluding nonreporting cases, which did not substantially alter the costs (<10%) (Supplementary File 6, Supplementary Digital Content 1, http://links.lww.com/CTG/B443). Third, recruiting patients through Dutch Celiac Society may have introduced selection bias because its members may not represent all persons with CD for GFD adherence and disease severity. Individuals who experienced debilitating symptoms before or after diagnosis may have been more motivated to participate in this study than asymptomatic individuals. Fourth, children's parents or guardians could serve as proxies, which may have resulted in over and/or under reporting of symptoms and QoL. Finally, health care utilization and costs associated with CD-related comorbidities (e.g., osteoporosis) might not have been considered by all respondents when completing the questionnaire, potentially underestimating costs.

The societal perspective used is a strength. Previous studies have examined either health care costs or productivity costs, whereas this is the first study to combine both alongside GFD costs (17–20). Adopting this societal perspective is increasingly recommended in economic evaluations because it safeguards against the omission of costs outside of the health sector that may represent major cost drivers (15,41). Moreover, a comparison of QoL and societal costs was made between children and adults, which, to our knowledge, has not been conducted before. Finally, our study sample, which included more than 2,600 patients with CD, was larger than those in previous studies on QoL (8–10).

This cross-sectional study showed that QoL improves significantly after CD diagnosis, and that health care and societal costs are significantly reduced, except for societal costs for those diagnosed during childhood, compared with recalled prediagnosis states. Our multivariate analyses highlighted several factors that may influence CD-related QoL and costs prediagnosis and postdiagnosis. Our findings contribute to a deeper understanding of the burden of CD and its societally financial impact, supporting efforts by healthcare professionals and policymakers to promote earlier identification of CD. Future research should explore the role of CD-associated comorbidities for their contribution/influence on patients' QoL and accrued costs and target more generally-representative populations for data collection.

## CONFLICTS OF INTEREST

**Guarantor of the article:** Anna L. Heilig, MSc.

**Specific author contributions:** None to report.

**Financial support:** None to report.

**Potential competing interests:** None to report.

**Study Highlights**

**WHAT IS KNOWN**

✓ Quality of life (QoL) for people with celiac disease (CD) is negatively affected by the intraintestinal and extraintestinal symptoms they experience.
✓ Adherence to a gluten-free diet (GFD) is currently the only effective treatment for CD. The restrictive nature and high cost of this GFD are reported barriers to compliance.
✓ Conflicting findings on CD-related health care use and productivity costs highlight a knowledge gap in understanding the economic burden of the disease.

**WHAT IS NEW HERE**

✓ QoL improved significantly after a CD diagnosis, with greater average improvements reported among individuals diagnosed during childhood.
✓ Annual health care and societal costs decreased after a CD diagnosis, except for societal costs among those diagnosed in childhood.
✓ Several factors were significantly associated with QoL, annual health care costs, and societal costs before and after diagnosis.

## Supplementary Material

## ABBREVIATIONS:

CD: celiac disease
EQ-VAS: EQ Visual Analogue Scale
GFD: gluten-free diet
GP: general practitioner
QoL: quality of life
