# Supplementary material for: Quality of Life and Societal Costs Related to Celiac Disease Before and After Diagnosis
Source: Clin Transl Gastroenterol. 2025 Dec 19;17(2):e00965. doi: 10.14309/ctg.0000000000000965 (PMC12922927; doi:10.14309/ctg.0000000000000965)
Supplement: Supplementary file 1 [file ct9-17-e00965-s001.docx]

**Supplementary legends**

**Supplementary 1 Questionnaire 1 – Glutenscreen DCS questionnaire (Qualtrix)**

**Supplementary 2 Table 1 – Pearson’s correlation covariates multivariate regressions**

**Supplementary 3 Table 2 – Prevalence symptoms/signs pre-diagnosis**

**Supplementary 4 Table 3 – Reported QoL pre- and post-diagnosis**

**Supplementary 5 Graphs & Tables – Time since diagnosis and EQ-utility, societal costs**

**Supplementary 6 Table – Healthcare, non-healthcare and societal costs excluding non-reporting cases**

**Supplementary 1 Questionnaire 1 – Glutenscreen DCS questionnaire (Qualtrix)**

Q1 Thank you for your interest in participating in this study. Participation is voluntary, and you may stop completing the questionnaire at any time. You do not need to provide a reason.

**What will happen to my data?**

By participating in the study, you are also giving your consent for your data to be collected, used, and stored. Rest assured that this research data is anonymous and can never be traced back to you.

*We store the following data:*

- your gender

- your age

- Information regarding your health

Q2 Do you agree to the use of your data for research purposes?

- Yes (1)
- No (2)

Q3 This questionnaire collects information about the impact of celiac disease on your life. It begins with general questions, followed by questions about the disease’s impact on your life before and after diagnosis.

Q4 How old are you?

- Years (e.g. 47) (1) __________________________________________________

Q5 What is your gender?

- Female (1)
- Male (2)
- Other (3)

Q6 When were you diagnosed with celiac disease?

- Year (e.g. 2001) (1) __________________________________________________

Q7 How long did your **symptoms last before receiving a diagnosis?** *Give your best estimate.*

- Years (mandatory) (1) __________________________________________________
- and months (optional) (2) __________________________________________________

Situation before diagnosis

Q46 The following questions are about your situation **prior to being diagnosed** with celiac disease.

|  |
| --- |

Q47 How many times did you visit the healthcare providers listed below for your symptoms **up until diagnosis** with celiac disease (including the consultation in which the diagnosis was made)? *If you did not visit a healthcare provider, select 0 times.*

|  |  |
| --- | --- |
| General practitioner (1) | ▼ 0 times (1) ... more than 10 times (12) |
| Internist (2) | ▼ 0 times (1) ... more than 10 times (12) |
| Gastroenterologist (3) | ▼ 0 times (1) ... more than 10 times (12) |
| Dietitian (4) | ▼ 0 times (1) ... more than 10 times (12) |
| Other, namely (5) | ▼ 0 times (1) ... more than 10 times (12) |
| Other, namely (6) | ▼ 0 times (1) ... more than 10 times (12) |

Q9 **Before** your celiac disease diagnosis, did you take time off work due to symptoms or scheduled visits with healthcare providers **related to celiac disease**?

- Yes (1)
- No (2)
- Not applicable (I did not work then) (3)

Q10 On average, how many **days per year** were you absent from work before your diagnosis? *If you don’t know exactly, give your best estimate.*

- Days per year (number) (1) __________________________________________________

| Q16 Which of the following symptoms did you experience **before your diagnosis** (continuous or intermittent). *If you did not have any symptoms, check the box at the bottom “None of the above”.* | Select (1) |
| --- | --- |
| Diarrhea (1) |  |
| Constipation (2) |  |
| Chronic fatigue (3) |  |
| Abdominal pain/fatigue (4) |  |
| Flatulence/excessive gas (5) |  |
| Headache (6) |  |
| Joint pain (7) |  |
| Osteoporosis (8) |  |
| Skin rash (9) |  |
| Mouth ulcers (10) |  |
| Anemia (11) |  |
| Depression (12) |  |
| Ataxia (13) |  |
| Other, namely (14) |  |
| None of the above (15) |  |

Part 2 (health after the diagnosis)

Q48 The next two questions ask you to consider your overall health before and after your celiac disease diagnosis. General health includes factors that are and are not directly related to celiac disease.

| Q18 Please indicate which statements best describe your health status **before diagnosis (left column)** and **your current situation after the diagnosis (right column).** | **Before celiac diagnosis**  (average day) (1) | **Current** average day   (2) |
| --- | --- | --- |
| I have no problem walking (1) |  |  |
| I’m having a little trouble walking (2) |  |  |
| I have moderate difficulty walking (3) |  |  |
| I have serious trouble walking (4) |  |  |
| I am unable to walk (5) |  |  |
| I have no problems washing or dressing myself (6) |  |  |
| I have a little trouble washing or dressing myself (7) |  |  |
| I have moderate difficulty washing or dressing myself (8) |  |  |
| I have serious trouble washing or dressing myself (9) |  |  |
| I am unable to wash or dress myself (10) |  |  |
| I have no problems with my daily activities (11) |  |  |
| I have a little trouble with my daily activities (12) |  |  |
| I have moderate difficulty with my daily activities (13) |  |  |
| I have serious trouble with my daily activities (14) |  |  |
| I am unable to do my daily activities (15) |  |  |
| I am not experiencing any pain or discomfort (16) |  |  |
| I’m experiencing a little pain or discomfort (17) |  |  |
| I have moderate pain or discomfort (18) |  |  |
| I am experiencing severe pain or discomfort (19) |  |  |
| I am experiencing extreme pain or discomfort (20) |  |  |
| I am not anxious or gloomy (21) |  |  |
| I am a little anxious or gloomy (22) |  |  |
| I am moderately anxious or gloomy (23) |  |  |
| I am very anxious or gloomy (24) |  |  |
| I am severely anxious or gloomy (25) |  |  |

Q19 We want to know how good or bad your health was/is on an average day before and after your diagnosis.
- These measurement scales range from 0 to 100. - **100 represents the best health** you can image. - **0 represents the worst health** you can imagine.  
Use the arrow to indicate how your health was **before (upper scale)** and **after your diagnosis (lower scale).**

|  | 0 | 10 | 20 | 30 | 40 | 50 | 60 | 70 | 80 | 90 | 100 |
| --- | --- | --- | --- | --- | --- | --- | --- | --- | --- | --- | --- |

| Your health **before diagnosis** () | 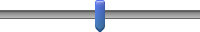 |
| --- | --- |
| Your **current** health () | 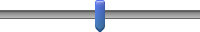 |

Part 3 (situation AFTER diagnosis)

Q45 The following questions concern your (current) situation **after being diagnosed with celiac disease**

Q20 Have you started a gluten-free diet?

- Yes, directly after diagnosis (1)
- Yes, some time after diagnosis (4)
- No (2)

Q21 When did you start a gluten-free diet?

- Year (e.g. 2001) (1) __________________________________________________

Q22 To what extent do you adhere to a gluten-free diet?

- Always (1)
- Frequently (2)
- Sometimes (about half the time) (3)
- Rarely (4)
- Never (5)

Q23 How often have you visited the healthcare providers listed below **in the past year** because of celiac disease? *If you have not visited a healthcare provider, select ‘0 times’.*

|  |  |
| --- | --- |
| General practitioner (1) | ▼ 0 times (1) ... more than 10 times (12) |
| Internist (2) | ▼ 0 times (1) ... more than 10 times (12) |
| Gastroenterologist (3) | ▼ 0 times (1) ... more than 10 times (12) |
| Dietitian (4) | ▼ 0 times (1) ... more than 10 times (12) |
| Other, namely (5) | ▼ 0 times (1) ... more than 10 times (12) |
| Other, namely (6) | ▼ 0 times (1) ... more than 10 times (12) |

Q25 Have you been absent from work **in the past year** due to celiac disease symptoms or related visits to healthcare providers?

- Yes (1)
- No (2)
- Not applicable (I am currently not working/no longer working) (3)

Q26 On average, how many **days** have you been absent from work duet o celiac disease symptoms or related visits to healthcare providers **in the past year?** *Please give your best estimate.*

- Days in the past year (number) (1) __________________________________________________

Q31 How was your celiac disease diagnosed? Check one or more options

|  | Select (1) |
| --- | --- |
| Blood test(s) (1) |  |
| Biopsy of the small intestine mucosa (sometimes performed by gastroscopy) (2) |  |
| Other, namely (3) |  |

Q32 Do you have any family members who have been diagnosed with celiac disease (excluding yourself)?

- If yes, how many (number) (1) __________________________________________________
- No (2)
- I don’t know (3)

Q34 Have you been diagnosed with any **other condition(s),** besides celiac disease?

- If yes, which conditions (1) __________________________________________________
- No (2)

Q36 Can you estimate the **additional weekly costs** you incur for food **due to** celiac disease? Please check the appilcable options.

- More than € 40 extra per week (1)
- between € 20-€ 40 extra per week (2)
- less than € 20 extra per week (3)
- No additional costs (4)

**Supplementary 2 Table 1 – Pearson’s correlation covariates multivariate regressions**

| Covariates^1^ | Sex (M/F) | Age at diagnosis (<18/≥18) | No. symptoms before diagnosis^2^ | Duration symptoms till diagnosis (yr)^2^ | Comorbidities (No/Yes) | GFD adh. (Always/Not) |
| --- | --- | --- | --- | --- | --- | --- |
| Sex (M/F) | -- | 0.01 | -0.01 | 0.01 | 0.04 | < 0.01 |
| Age at diagnosis (<18/≥18) | 0.01 | -- | 0.16 | 0.29 | 0.21 | 0.01 |
| No. symptoms before diagnosis^2^ | -0.01 | 0.16 | -- | 0.36 | 0.17 | -0.03 |
| Duration symptoms till diagnosis (yr)^2^ | 0.01 | 0.29 | 0.36 | -- | 0.14 | -0.01 |
| Comorbidities (No/Yes) | 0.04 | 0.21 | 0.17 | 0.14 | -- | -0.01 |
| GFD adh. (Always/Not) | < 0.01 | 0.01 | -0.033 | -0.01 | -0.01 | -- |

^1^Sample size: 2683; ^2^Variables were reported retrospectively;

Note: If the correlation between two variables exceeded moderate correlation (R^2^ > 0.60), a variable was excluded from the regression analyses.

**Supplementary 3 Table 2 – Prevalence symptoms/signs pre-diagnosis^2^**

| **Symptoms, N (%)^1^** | **Overall** | **< 18 years** | **≥18 years** |
| --- | --- | --- | --- |
| *Any symptom* | 2628 (98%) | 575 (97%) | 2053 (98%) |
| *Abdominal pain/bloating* | 1964 (73%) | 471 (79%) | 1493 (71%) |
| *Chronic fatigue* | 1801 (67%) | 369 (62%) | 1432 (68%) |
| *Diarrhea* | 1583 (59%) | 333 (56%) | 1250 (60%) |
| *(Excessive) flatulence* | 1329 (49%) | 188 (32%) | 1141 (55%) |
| *Anemia* | 1034 (38%) | 128 (22%) | 906 (43%) |
| *Constipation* | 1006 (37%) | 227 (38%) | 779 (37%) |
| *Headache* | 835 (31%) | 140 (24%) | 695 (33%) |
| *Skin rashes* | 647 (24%) | 124 (21%) | 523 (25%) |
| *Joint pain* | 593 (22%) | 47 (8%) | 546 (26%) |
| *Depression* | 514 (19%) | 51 (9%) | 463 (22%) |
| *Mouth ulcer* | 512 (19%) | 64 (11%) | 448 (21%) |
| *Osteoporosis* | 216 (8%) | 4 (0.7%) | 212 (10%) |
| *Ataxia* | 46 (2%) | 4 (0.7%) | 42 (2%) |
| *Other symptoms* | 655 (24%) | 228 (38%) | 427 (20%) |

^1^Sample size: 2691; ^2^Participants retrospectively reported symptoms;

**Supplementary 4 Table 3 – Reported QoL pre- and post-diagnosis**

| **EQ-5D-5L domain^1^** | **Before CD diagnosis^2^** | **After CD diagnosis** |
| --- | --- | --- |
| Mobility, N(%)   1. No problems 2. Slight problems 3. Moderate problems 4. Severe problems 5. Extreme problems | 2172 (81%)  242 (9%)  152 (6%)  84 (3%)  35 (1%) | 2275 (85%)  231 (9%)  133 (5%)  44 (2%)  2 (0.1%) |
| Self-care, N(%)   1. No problems 2. Slight problems 3. Moderate problems 4. Severe problems 5. Extreme problems | 2476 (92%)  96 (4%)  42 (2%)  30 (1%)  41 (1%) | 2582 (96%)  71 (3%)  23 (1%)  4 (0.1%)  5 (0.2%) |
| Usual activities, N(%)   1. No problems 2. Slight problems 3. Moderate problems 4. Severe problems 5. Extreme problems | 1111 (41%)  553 (21%)  575 (21%)  366 (14%)  80 (3%) | 1808 (67%)  548 (20%)  242 (9%)  76 (3%)  11 (0.4%) |
| Pain / discomfort, N(%)   1. No problems 2. Slight problems 3. Moderate problems 4. Severe problems 5. Extreme problems | 587 (22%)  519 (19%)  798 (30%)  619 (23%)  162 (6%) | 1354 (50%)  914 (34%)  322 (12%)  88 (3%)  7 (0.3%) |
| Anxiety / depression, N(%)   1. No problems 2. Slight problems 3. Moderate problems 4. Severe problems 5. Extreme problems | 1273 (47%)  558 (21%)  500 (19%)  279 (10%)  75 (3%) | 1692 (63%)  693 (26%)  225 (8%)  60 (2%)  15 (0.6%) |

^1^Sample size: 2685; ^2^Participants retrospectively reported pre-diagnosis QoL;

**Supplementary 5 Graphs & Tables – Time since diagnosis and EQ-utility, societal costs**

*Quality of life:*


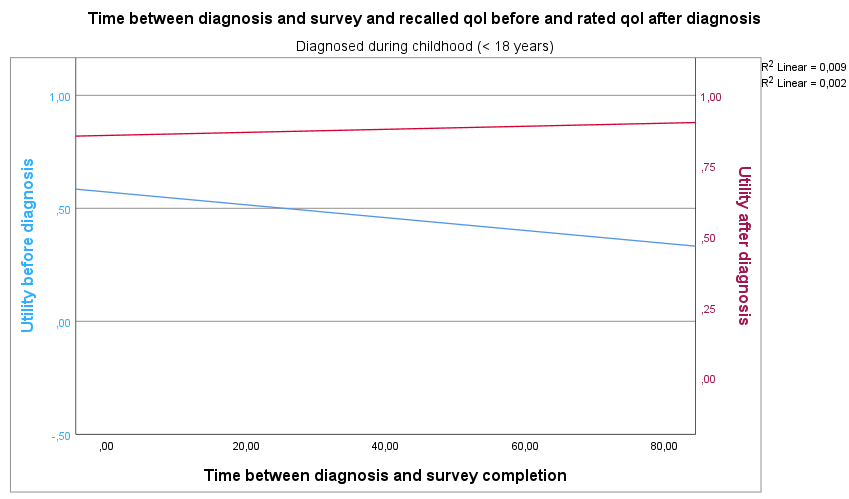


Graph 1 – Association between time since diagnosis and pre- and post-diagnosis EQ-utility for child diagnoses


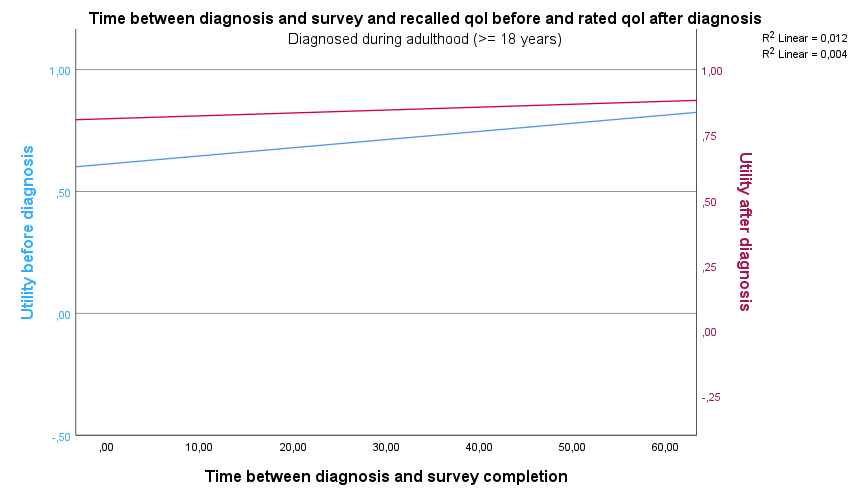


Graph 2 – Association between time since diagnosis and pre- and post-diagnosis EQ-utility for adult diagnoses


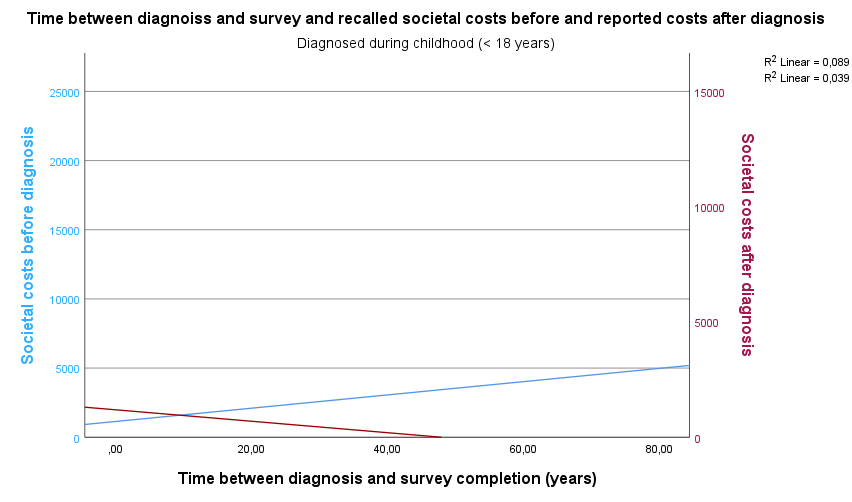


Graph 3 – Association between time since diagnosis and pre- and post-diagnosis societal costs for child diagnoses


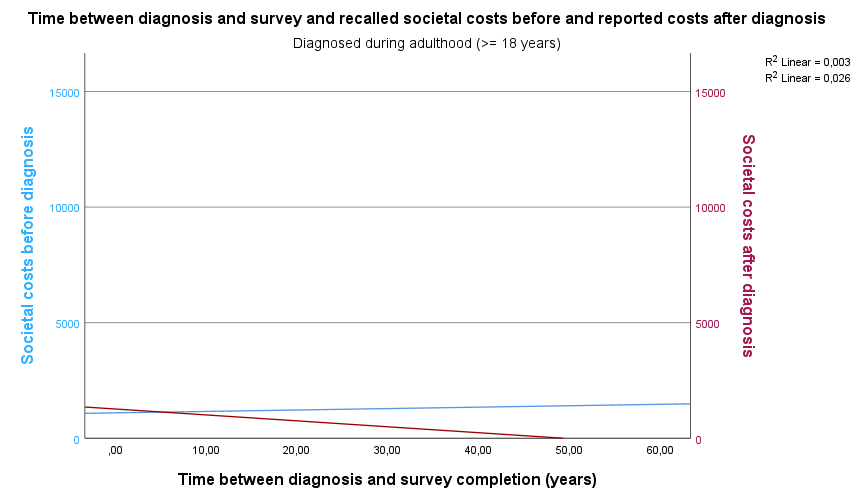


Graph 4 – Association between time since diagnosis and pre- and post-diagnosis societal costs for adult diagnoses

**Table – linear regression time between diagnosis and survey completion and utility, societal costs^1^**

| **Variables** | **< 18 years** | | **≥ 18 years** | |
| --- | --- | --- | --- | --- |
|  | **Constant** | **Coefficient (95% CI)** | **Constant** | **Coefficient (95% CI)** |
| Pre-utility | 0.596 | -0.003* (-0.006 : -0.001) | 0.624 | 0.003* (0.002 : 0.004) |
| Post-utility | 0.859 | 0.001 (-0.001 : 0.001) | 0.811 | 0.001* (0.000 : 0.002) |
| Pre societal costs | 3228 | 8 (-48 : 65) | 6030 | 59 (-7 : 126) |
| Post societal costs | 4075 | -62* (-95 : -29) | 5275 | -137* (-175 : -99) |

*Significant (p < 0.05); ^1^Sample size: 2685;

**Supplementary 6 Table – Healthcare, non-healthcare and societal costs excluding non-reporting cases**

**Table - Percentage difference in healthcare, non-healthcare, and societal cost data with and without estimation^1^**

| **Costs** | Before diagnosis (B), *mean (% difference)* | | | After diagnosis (A), *mean (% difference)* | | |
| --- | --- | --- | --- | --- | --- | --- |
|  | B; overall | B; < 18 | B >= 18 | B; overall | A: < 18 | A: >= 18 |
| General practitioner costs | 236 (+3.1%) | 367 (+5.2%) | 200 (+2.6%) | 74 (+2.8%) | 57 (+5.6%) | 79 (+1.3%) |
| Internist costs | 162 (+5.9%) | 120 (+8.1%) | 174 (+6.1%) | 91(+1.1%) | 35 (-2.8%) | 106 (+1.0%) |
| Gastroenterologist costs | 297 (0%) | 399 (+0.8%) | 269 (0%) | 411 (+0.7%) | 248 (+0.4%) | 455 (+0.2%) |
| Dietician costs | 96 (+6.7%) | 104 (+8.3%) | 93 (+5.7%) | 251 (+1.2%) | 240 (+1.2%) | 254 (+1.2%) |
| Other resource costs | 193 (+6.0%) | 526 (+8.0%) | 102 (+6.3%) | 165 (-0.1%) | 363 (+2.0%) | 111 (-1.8%) |
| **Healthcare costs** | 1302 (+2.7%) | 1722 (+4.6%) | 1187 (+2.2%) | 992 (+0.8%) | 943 (+1.5%) | 1005 (+0.5%) |
| Productivity losses^2^ | 4888 (+5.2%) | 1795 (+7.5%) | 5739 (+4.7%) | 1338 (+5.4%) | 957 (+6.8%) | 1443 (+4.9%) |
| GFD costs | - |  |  | 1506 (-0.1%) | 1599 (+0.4%) | 1481 (-0.1%) |
| **Non-healthcare costs^1^** | 4888 (+5.2%) | 1795 (+7.5%) | 5739  (+4.7%) | 2844 (+2.4%) | 2555 (+2.7%) | 2924 (+2.3%) |
| **Societal costs** | 6190 (+4.7%) | 3517  (+6.1%) | 6926  (+4.2%) | 3836 (+2.0%) | 3498 (+2.4%) | 3929 (+1.9%) |

*^1^2685 respondents;*
